# Effects of Polyphenols from Oat and Oat Bran on Anti-Inflammatory Activity and Intestinal Barrier Function in Raw264.7 and Caco-2 Models

**DOI:** 10.3390/nu17121962

**Published:** 2025-06-09

**Authors:** Wen Duan, Bisheng Zheng, Tong Li, Rui Hai Liu

**Affiliations:** 1 School of Food Sciences and Engineering, South China University of Technology, Guangzhou 510641, China; 15754367187@163.com (W.D.); febzheng@scut.edu.cn (B.Z.); 2 Overseas Expertise Introduction Center for Discipline Innovation of Food Nutrition and Human Health, School of Food Sciences and Engineering, South China University of Technology, Guangzhou 510641, China; 3 Department of Food Science, Cornell University, Ithaca, NY 14853, USA; tl24@cornell.edu

**Keywords:** oats, bran, polyphenols, inflammation, intestinal barrier

## Abstract

Background/Objectives: Oats and oat bran are rich in dietary fiber, polyphenols and other phytochemicals. Methods: In this study, we evaluated the phytochemical content and established LPS-induced RAW 264.7 macrophage inflammation and DSS-induced Caco-2 cell inflammation models to investigate the anti-inflammatory activities of oat and oat bran polyphenols and their molecular mechanisms. Results: The results showed that oat and oat bran polyphenols (free and bound polyphenols) enhanced phagocytosis, decreased the expression of nitric oxide synthase (iNOS) and cyclooxygenase-2 (COX-2), reduced the production of NO and ROS, increased the mitochondrial membrane potential, and reduced the inflammatory cytokines (TNF-α, IL-1β, and IL-6) at the gene level in the RAW 264.7 macrophage inflammation model induced by LPS expression, thus demonstrating strong anti-inflammatory activity. In Caco-2 cells, oat and oat bran polyphenols pretreatment attenuated the DSS-induced decrease in trans-epithelial electron resistance value, increased tight junction protein expression, and reduced cell permeability in Caco-2 cell monolayers, which in turn reduced inflammatory damage in the organism. Conclusions: In summary, the present study not only reveals the mechanism by which oat and oat bran polyphenols inhibit macrophage inflammation and impairment of intestinal barrier function at defined concentration in vitro, but also highlights potential for oat bran as a functional food.

## 1. Introduction

Inflammation is a complex protective physiological response to the immune defense mechanism that occurs when the body is exposed to external stimuli or injury [1]. The inflammatory response is a fundamental immune reaction that maintains tissue homeostasis. However, abnormalities in this process can lead to various diseases, such as irritable bowel syndrome and Parkinson’s disease [2]. Prolonged metabolic chronic low-grade inflammation can lead to various metabolic disorders. Currently, several drugs are used clinically to treat inflammation and can exhibit anti-inflammatory effects to some extent. However, long-term use of these drugs can lead to side effects and other issues [3]. While functional foods are not a replacement for clinical drug therapy, they can serve as a nutritional intervention to help mitigate chronic inflammatory responses. Therefore, the research and development of new, safe, and non-toxic anti-inflammatory health foods or drugs derived from natural plants have become major focal points in the field of life sciences.

The health of the human gut is of paramount importance. The gut is not only a major organ for digestion, absorption, and metabolism, but it also serves as a crucial physical, chemical, immune, and microbial barrier [4]. The intestinal barrier is the body’s first line of defense against luminal microorganisms and food antigens. Structural damage to this barrier can allow harmful substances, such as toxins and pathogenic bacteria, to enter the circulatory system, initiating an immune response that can result in localized inflammation or systemic disease [5]. Abnormalities in the barrier function of the intestinal mucosa are significant causes of inflammatory bowel disease [6], intestinal stress syndrome, and intestinal tumorigenesis. The structure of the intestinal epithelium, composed of intestinal epithelial cells and intercellular junctions, is crucial for preventing the invasion of pathogenic microorganisms and bacterial translocation. Therefore, improving the function of the intestinal epithelium and maintaining the integrity of the intercellular tight junctions will help mitigate the inflammatory response [7].

A growing body of research suggests that the disruption of gut barrier may lead to disease onset or accelerated disease progression. Loss of barrier integrity can increase the translocation of bacterial antigens, stimulate intestinal mucosal inflammation, and trigger the onset of intestinal disease [8]. Studies have shown that some functional components in cereals can inhibit or alleviate inflammation in the body without toxic side effects [9,10]. Additionally, the abundant polyphenolic compounds in cereals can scavenge a large number of reactive oxygen species in the body due to their excellent antioxidant activity, thus preventing the occurrence of diseases [11,12].

Oats are a traditional cereal food that can regulate the human gut microbiota, thus positively affecting health [13]. Oat bran, the main by-product of oat processing, has high nutritional value and is rich in dietary fiber. However, a large amount of oat bran is currently used as feed, leading to low utilization and economic benefits. It is well known that oats and oat bran are rich in β-glucans and phenolic acids, which have antioxidant, anti-inflammatory, and gut health-regulating effects [14,15,16].

It has been reported that about 60–85% of polyphenols in wheat bran exist in bound polyphenols, and bound polyphenols in grains exhibit high biological activities such as antioxidant and antihyperglycemic properties [17,18]. The experimental results of Chen et al. (2019) [19] demonstrated that the in vitro digestion process significantly reduced the concentration of polyphenols in oats. The degradation of polyphenols primarily occurs in the intestinal tract, where they undergo absorption and transit during the digestive process [19]. Li et al. (2022) found that highland barley attenuates inflammation and intestinal dysbiosis induced by a high-fat diet by inhibiting inflammatory factors and reestablishing the gut microbiome and lipid metabolism [20]. Hong et al. (2020) used LPS-induced RAW264.7 cells to establish inflammation model to evaluate the anti-inflammatory activity of sorghum polyphenols and showed that sorghum polyphenol extracts significantly reduced NO and ROS levels in cells [21]. Numerous studies have demonstrated the efficacy of polyphenolic substances in natural products in modulating intestinal inflammation [22].

Plant flavonoids can protect barrier integrity by acting on intestinal tight junction structures and maintaining mucosal homeostasis. Human colon cancer Caco-2 cells are a classic cell line used to study the secretion of intestinal mucosal epithelial cells and the function of intestinal barrier damage in vitro [23]. Kim et al. used a lipopolysaccharide-stimulated Caco-2/RAW264.7 co-culture model to determine the effects of acai berries on intestinal inflammation. The results showed that the addition of acai berries restored the epithelial resistive value, inhibited IL-8 and IL-6 mRNA expression in Caco-2 cells, and restored cell barrier function, thereby improving intestinal health [24]. In conclusion, the exact mechanism by which oat and bran polyphenols modulate macrophage inflammation and the intestinal cell barrier is still unknown.

Therefore, in this study, we established an LPS-induced inflammation model of RAW264.7 cells and a DSS-induced inflammation model of Caco-2 cells in vitro. We explored the protective effects of polyphenols from oats and bran on macrophage inflammation and barrier damage in Caco-2 cells by determining the expression of inflammatory factors and the level of tight junction proteins. Our aim was to elucidate the mechanism of the anti-inflammatory efficacy of oats and oat bran and provide theoretical references for the development and utilization of oats and bran as precision nutritional foods.

## 2. Materials and Methods

### 2.1. Materials and Reagents

Fluorescein sodium salt, dichlorofluorescin diacetate (DCFH-DA) and lipopolysaccharide (LPS) (*Escherichia coli*, serotype 0111:04) were purchased from Sigma-Aldrich Chemical Co. (St. Louis, MO, USA). The NO assay kit, neutral red, high-efficiency RIPA cell lysis solution was purchased from Beijing Solepol Technology Co. (Beijing, China). The oat and oat bran samples were provided by Yangufang Group (Inner Mongolia, Wuchuan, China).

### 2.2. Sample Preparation

Polyphenol was obtained according to the method of Adom [11] (Adom & Liu, 2002). Free polyphenol: An amount of 20 g of oat flour was mixed with 80% chilled acetone (1:10, *w*/*v*), homogenized using Virtis High Speed Homogenizer in 12,000× *g* for 5 min, and centrifuged at 4 °C, 10,000 r/min for 10 min to collected the supernatant. Repeat 3 times. All supernatants were combined and evaporated to dryness by vacuum rotary concentration at 45 °C and further reconstituted with 20 mL of 80% methanol for standby. Bound polyphenol: Add 2 M NaOH to the residue after extraction, shake for 1 h, and then adjust the pH of the reaction to 2 with concentrated HCl. The lipids were removed by 2 times extraction with n-hexane, followed by adding ethyl acetate to collected bound polyphenol. The extraction was repeated 6 times. The organic solution was combined, concentrated by vacuum rotation, and then volumetized to 20 mL by 80% methanol and stored at −40 °C for subsequent experiments.

### 2.3. Phytochemical Profiles Analysis by HPLC

Phytochemical profiles of oats was obtained using the method of He et al. (2022) [25] with modifications. The HPLC system (Waters Corporation, Milford, MA, USA) was used to analyze standard products. The determination conditions were based on the method of He et al. (2022) [25].

### 2.4. Cell Culture and Viability

Human colon cancer Caco-2 cells and RAW 264.7 cells were obtained from ATCC Co. (Manassas, VA, USA). The cell generations were in the range of 10–20, and the medium was changed every two days.

RAW 264.7 cells were spread in 96-well plates (1 × 10^4^ cells/well) and incubated for 24 h. Subsequently, 1 μg/mL LPS and different concentrations of polyphenol samples were added to the cells for 24 h. After the incubation was completed, the cell viability of each group was determined using the MTT assay. Caco-2 cells were spread in 96-well plates (3 × 10^4^ cells/well), pretreated with polyphenols for 24 h, and then stimulated with 1.5% DSS for 24 h. The cell viability of each group was determined using the MTT assay [26].

### 2.5. NO Assay

**Figure A1:**
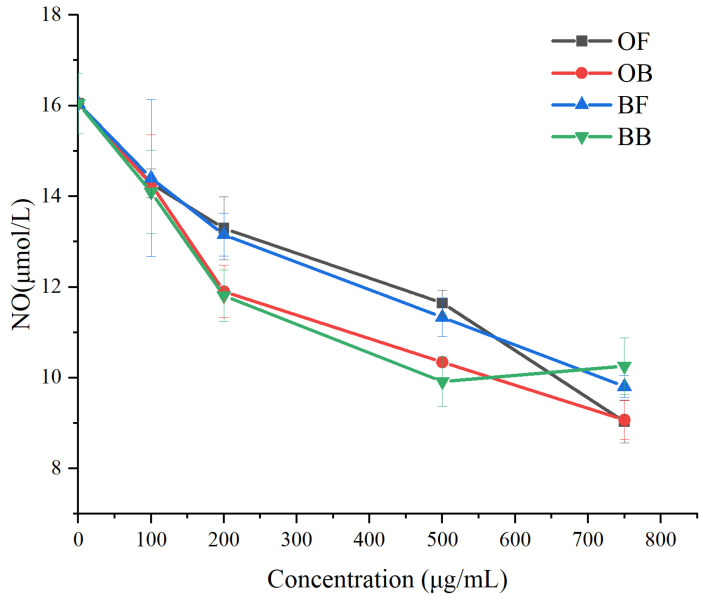
Effects of different concentrations of oat and oat bran polyphenols on NO formation in Raw 264.7 macrophages. OF (oat free polyphenol); BF (bran free polyphenol); OB (oat bound polyphenol) and BB (oat bran bound polyphenol).

The determination of NO was carried out according to the Biyuntian NO detection kit [26]. During the pre-experimental stage, we first evaluated the NO inhibition efficiency at a concentration of 100 μg/mL using 96-well plates (see Figure A1). Based on the experimental results, a concentration range of 200–750 μg/mL was selected as the gradient for the formal experiments. RAW 264.7 cells were spread in 12-well plates (1 × 10^5^ cells/well) and incubated in an incubator for 24 h. Then, different concentrations of polyphenol samples were incubated for 6 h and treated with 1 μg/mL LPS for 24 h. The supernatant was collected, and the absorbance was measured.

### 2.6. Phagocytosis Assay

The method of Xiong et al. (2018) [27] was referred to and slightly modified. RAW264.7 cells were cultured, and the inflammatory response was stimulated as described in Section 2.2, followed by incubation for 24 h. The cells were divided into 14 groups: blank control group, LPS-treated group, oat free phenol low-, medium-, and high-dose groups, oat bound polyphenol low-, medium-, and high-dose groups, bran free phenol low-, medium-, and high-dose groups, and bran bound polyphenol low-, medium-, and high-dose groups. After incubation for 24 h, the supernatant was discarded, 100 μL of 0.05% neutral red solution was added for staining, and the staining solution was aspirated after 1 h. After washing with PBS three times, 150 μL of cell lysate was added and then measured at a wavelength of 550 nm after 15 min of oscillation.

### 2.7. ROS Assay

The determination of ROS was carried out from our established protocol with slight modifications [28]. RAW 264.7 cells (1.0 × 10^4^ cells/well) were inoculated in 96-well black plates for 24 h. Following 6 h polyphenol pretreatment, LPS (1 μg/mL) was added and incubated for 24 h. The medium without samples was used as a control. Then, the fluorescence intensity was detected immediately after adding DCFH-DA solution (50 μmol) to the 96-well plate. The assay conditions were as follows: 37 °C, detection wavelength: 485 nm, emission wavelength: 538 nm, and the fluorescence intensity was measured every 5 min for 2 h. The fluorescence intensity was measured at the same time.

### 2.8. Mitochondrial Membrane Potential Assay

Referring to the method of Kapoor et al. (2023) [29] with appropriate modifications, RAW 264.7 cells were inoculated at a density of 4 × 105 cells and incubated for 24 h. The cells were treated with polyphenols for 6 h, and then LPS (1 μg/mL) was added and incubated for 24 h. After aspirating the supernatant, 1 mL of JC-1 staining working solution was added and mixed thoroughly. After the cells were incubated at a constant temperature of 37 °C for 20 min, the culture supernatant was discarded and washed three times using JC-1 staining buffer. The processed cells were subsequently resuspended in 2 mL of fresh culture solution for fluorescence microscopic observation.

### 2.9. Establishment of the Caco-2 Monolayer Model

The monolayer membrane model of Caco-2 cells was established with reference to the method of Yang et al. (2022) [30]. Cells were inoculated in the upper chamber of Transwell plates with 1.5 mL of complete medium in the lower chamber, and the blank group was given complete medium (0.5 and 1.5 mL) in both the upper and lower chambers. The culture medium was changed every two days for the first week and then every day until the culture reached 21 d. The monolayer formation of Caco-2 cells was assessed using the following three methods.

(1) The transmembrane electrical resistance (TEER) of cell membranes was measured using a resistivity meter to assess the integrity and tightness of the monolayers. The TEER of the cell membranes was measured every two days, and Caco-2 monolayers with a TEER value exceeding 500 Ω · cm^2^ were used for subsequent experiments. The formula is as follows:

TEER = (R1 − R2) × A (Ω · cm^2^)

R1 (Ω) is the resistance measurement of the Caco-2 cell compartment, R2 (Ω) is the resistance measurement of the blank control group, and A (cm^2^) represents the membrane area of the compartment.

(2) The polarity of the Caco-2 cell monolayer membrane can be assessed by measuring the ratio of alkaline phosphatase (ALP) activity between the upper and lower compartments of Transwell plates. The monolayer polarity of the Caco-2 cell model was tested by aspirating the culture fluid from the upper (AP) and lower (BL) chambers of the Transwell plate every two days, determining their ALP activities, and calculating the ratio of AP side to BL side.

(3) Caco-2 cell monolayer integrity was detected by measuring the passive diffusion of sodium fluorescein. Standard solutions of sodium fluorescein at concentrations of 0.5, 1, 2, 4, 6, 8, and 10 µg/mL were prepared with HBSS buffer, and the fluorescence intensity was measured using a multifunctional enzyme marker to generate a standard curve. When Caco-2 cells were cultured on Transwell plates for 21 d, the AP side and BL side of the chambers were washed three times using HBSS buffer; HBSS buffer was added, and after equilibrating in the incubator for 30 min, 0.5 mL of sodium fluorescein solution was added to the AP side, while 1.5 mL of HBSS buffer was added to the BL side. The chambers were cultured in CO_2_ incubator, and 100 µL of BL buffer was taken from the chambers at 60, 120, and 180 min. A total of 100 µL of BL side transporter solution was taken, the fluorescence intensity was measured by molecular ID3, and the concentration of sodium fluorescein was calculated according to the standard curve. The formula for calculating the transmittance of sodium fluorescein is as follows:

transmission rate (%) = (Mass of sodium fluorescein on the BL side)/(Mass of sodium fluorescein on the initial AP side) × 100

### 2.10. Cytokine Assays

The levels of IL-6, IL-10, and TNF-α in the supernatant were determined using an ELISA kit (Multi Sciences Biotech Co., Ltd., Hangzhou, Zhejiang, China). Caco-2 cells were pretreated with different concentrations of polyphenols for 24 h and then stimulated with 1.5% DSS for 24 h. The measurement of IL-6 and IL-8 in the supernatants was conducted.

### 2.11. Real-Time PCR

The relative transcription levels of inflammatory mediators were determined by the qPCR analysis according to our reported method [31]. RAW 264.5 cells were inoculated at 4 × 10^5^ cells/mL. After 24 h, the cells were pretreated with oat and bran polyphenols for 6 h, followed by stimulation with LPS (1 μg/mL) for 24 h. Cell grouping refer to 2.4. At the end of culture, cells were rinsed twice with PBS after medium removal. Total RNA extraction was then performed through direct lysis using 500 μL TRIzol reagent per well, followed by reverse transcription to generate cDNA templates for subsequent qPCR amplification. The primers used were as follows: GAPDH, (F:CTCGTCCCGTAGACAAAATGGT,R:GAGGTCAATGAAGGGGTCGTT); iNOS, (F:CCTTACGAGGCGAAGAAGGACAG,R:CAGTTTGAGAGAGGAGGCTCCG); COX2, (F:GAGAGATGTATCCTCCCACAGTCA,R:GACCAGGCACCAGACCAAAG); TNF-α, (F:CAGGCGGTGCCTATGTCTC,R:CGATCACCCCGAAGTTCAGTAG); IL-6, (F:CTGCAAGAGACTTCCATCCAG,R:AGTGGTATAGACAGGTCTGTTGG); Trl4, (F:CAACATCATCCAGGAAGGC,R:GAAGGCGATACAATTCCACC). Caco-2 cells Sample processing for the Caco-2 cells model was referred to the steps described in Section 2.10, and at the end of the intervention, RNA was extracted separately. The primer sequences are as follows: GAPDH (F: TCCACTGGCGTCTTCACCACCAT, R: GGAGGCATTGCTGATGATCTTGAGG); Occludin (F: ATGAGACAGACTACACAACTGG, R: TTGTATTCATCAGCAGCAGCAGC). The relative expression of target genes was calculated using the 2^−ΔΔCT^ method.

### 2.12. Statistical Analysis

Method analysis and Tukey’s test were used to evaluate the correlation between the data (SPSS version 19), significance analysis *p* ˂ 0.05. All tests were repeated 3 times, and the test data were expressed as mean ± standard deviation.

## 3. Results

### 3.1. Phenolic Profiles

The content of phenolic acids in oat varieties and products is shown in Table A1. Eight phenolic compounds were detected in oat, namely gallic acid, protocatechuic acid, p-Hydroxybenzoic acid, vanillic acid, caffeic acid, p-Coumaric acid, ferulic acid and avenanthramides C. The ferulic acid content of the oat and bran ranged from 5.26 to 335.15 μg/g DW. Compared with oats, the content of ferulic acid (335.15 ± 1.64 μg/g DW) in bound polyphenol and Avenanthramides C (8.82 ± 4.04 μg/g DW) in free form in oat bran is higher. Ferulic acid is the most abundant phenolic substance and exists both as free and bound polyphenol, primarily in the bound form.

### 3.2. Effect of Oat and Oat Bran Polyphenols on Cell Viability

**Figure 1:**
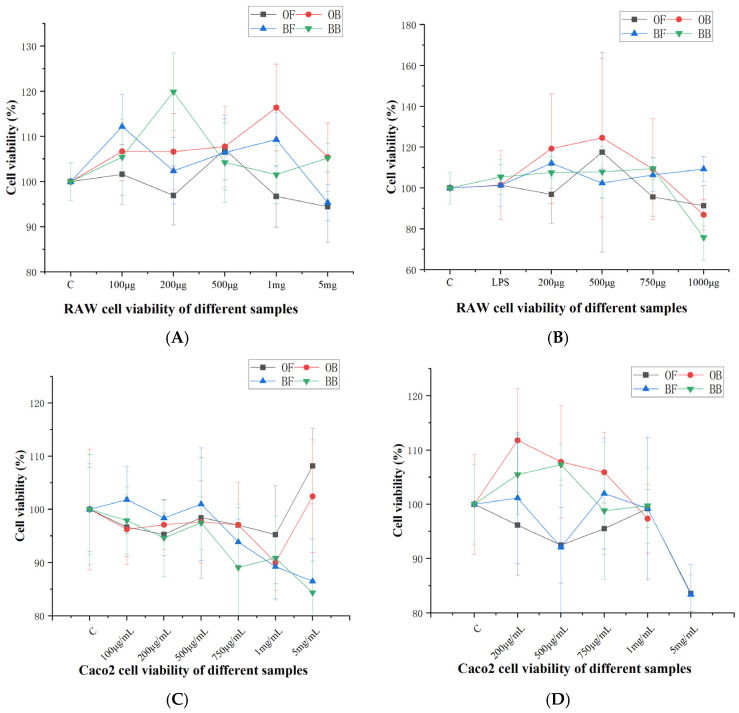
Effects of oat and bran polyphenol on Raw264.7 cell viability (**A**), in LPS-induced Raw264.7 cell viability (**B**), on Caco-2 cell viability (**C**), in DSS-induced Caco-2 cell viability (**D**). OF (oat free polyphenol); BF (bran free polyphenol); OB (oat bound polyphenol); BB (oat bran bound polyphenol).

The effects of different concentrations of oat and oat bran polyphenols on RAW 264.7 and Caco-2 cell viability after 24 h of intervention are shown in Figure 1. In Figure 1A, oat polyphenols and oat bran polyphenols showed no cytotoxic effects on cells across concentrations ranging from 0 to 5 mg/mL, respectively. In Figure 1B, compared with the blank group, none of the samples had cytotoxic effects at 1000 μg/mL concentration, except for the LPS-stimulated oat bran bound polyphenol (BB) (1000 μg/mL), which exhibited toxicity after 24 h of stimulation. Therefore, oat free polyphenol (OF) (200, 500, 750 μg/mL), oat bound polyphenol (OB) (200, 500, 750 μg/mL), oat bran free polyphenol (BF) (200, 500, 750 μg/mL), and oat bran bound polyphenol (BB) (200, 500, 750 μg/mL) were selected as the concentration gradients for subsequent tests. In Figure 1C, we evaluated the toxicity of oat and oat bran polyphenols on Caco-2 cells. After treatment with oat and bran polyphenols for 24 h, oat polyphenols did not show toxic effects up to a concentration of 5 mg/mL, whereas oat bran polyphenols inhibited the growth of Caco-2 cells at a concentration of 5 mg/mL, producing toxic effects. Following treatment with 1.5% DSS, both polyphenols did not produce toxicity at a concentration of 1 mg/mL. Therefore, the maximum dose examined (1 mg/mL) was selected to treat Caco-2 cells in subsequent experiments.

### 3.3. Effect of Oat and Oat Bran Polyphenols on Phagocytosis of RAW264.7 Cells

**Figure 2:**
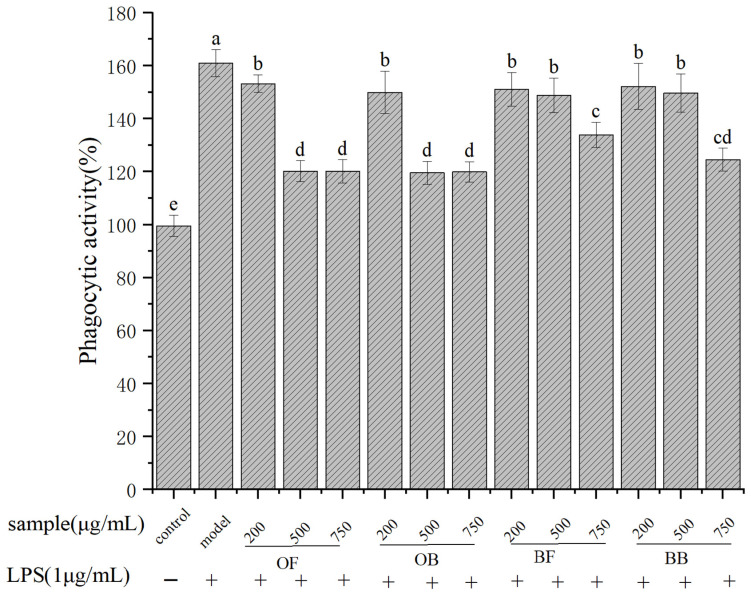
Effect of oat and bran polyphenols in LPS-induced phagocytosis of Raw264.7 macrophages cells. OF (oat free polyphenol); BF (bran free polyphenol); OB (oat bound polyphenol); BB (oat bran bound polyphenol). Different lowercase represent significant differences among groups.

In Figure 2, LPS stimulation significantly stimulated macrophages phagocytosis of neutral red after 24 h of stimulation, increasing phagocytosis compared to the blank group. However, after pretreatment with 200, 500, and 750 μg/mL oat and oat bran polyphenols, LPS-induced phagocytosis of mouse macrophages was significantly inhibited, with the highest inhibition observed at high doses.

### 3.4. NO and ROS Production

**Figure 3:**
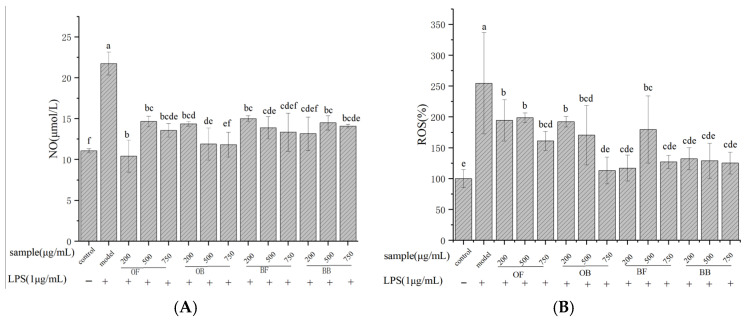
Effects of oat and bran polyphenol on NO (**A**) and ROS (**B**) in Raw 264.7 macrophages cell. OF (oat free polyphenol); BF (bran free polyphenol); OB (oat bound polyphenol); BB (oat bran bound polyphenol). Different lowercase represent significant differences among groups.

As shown in Figure 3A, after 24 h of LPS stimulation, the secretion of NO from RAW 264.7 cells in the control group was low, while NO and ROS release in the model group were significantly higher than those in the blank group, indicating successful establishment of the inflammatory cell model. All concentrations of oat polyphenols and oat bran polyphenols significantly reduced NO release compared to the model group. The inhibition of NO secretion was most pronounced at concentrations of 750 μg/mL for oat free phenol, oat bound polyphenol, and oat bran free phenol, reducing NO production rates by 37.57%, 45.63%, and 38.62%, respectively. In Figure 3B, the relative expression of ROS after pretreatment with different concentrations of oat polyphenols and oat bran polyphenols ranged from 113.25% to 198.73%. Intracellular ROS release was significantly lower than that in the LPS group, and the inhibitory effect of high concentrations of polyphenols on ROS release was the strongest.

### 3.5. Inflammatory Cytokine Expression in Cells

**Figure 4:**
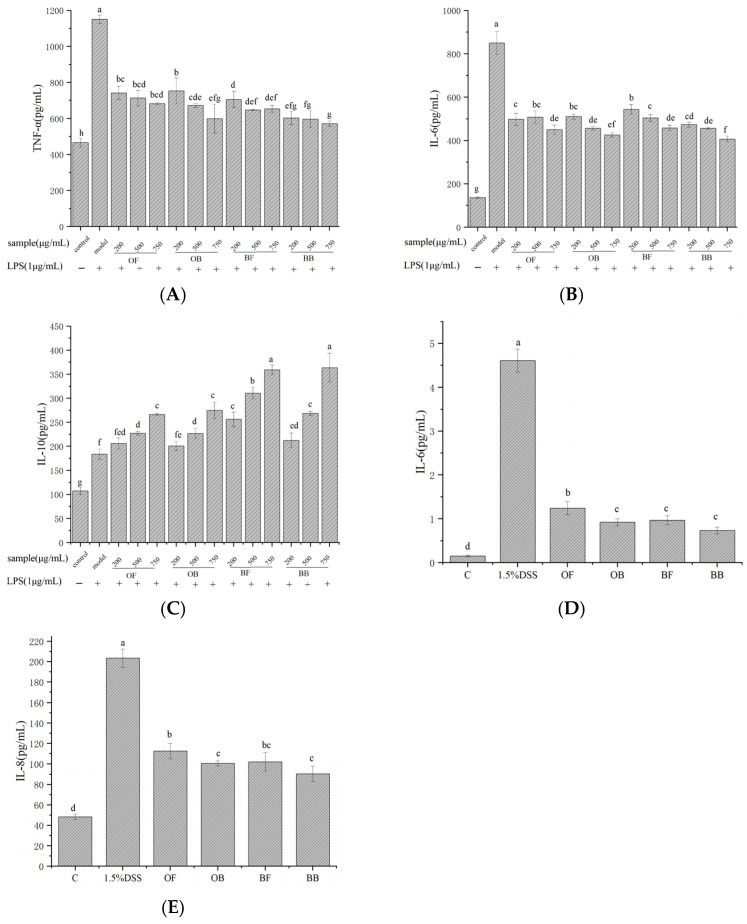
Effects of oat and bran polyphenol on the production of TNF-α (**A**), IL-6 (**B**), and IL-10 (**C**) in LPS-induced Raw264.7 macrophages and on the production of IL-6 (**D**) and IL-8 (**E**) in DSS-induced Caco-2 cell. OF (oat free polyphenol); BF (bran free polyphenol); OB (oat bound polyphenol); BB (oat bran bound polyphenol). Different lowercase represent significant differences among groups.

We determined the inhibitory effects of oat and oat bran polyphenols on the secretion of IL-6, TNF-α, and IL-10. In Figure 4A–C, in the model group, RAW 264.7 cells secreted significantly higher levels of pro-inflammatory factors IL-6 and TNF-α compared to the blank group, indicating successful induction of an inflammatory response by LPS in RAW 264.7 cells. Compared to the model group, the polyphenol-treated groups showed significant inhibition of pro-inflammatory factor secretion, with the level of secretion gradually decreasing with increasing treatment concentration. The secretion of the anti-inflammatory factor IL-10 exhibited a certain concentration dependence on increasing polyphenol treatment concentration, particularly showing significant enhancement at a concentration of 750 μg/mL. These findings further indicate that oat and bran polyphenols can alleviate the inflammatory response of macrophages induced by LPS and exhibit potent in vitro anti-inflammatory activity.

As shown in Figure 4D,E, compared to the blank group, DSS treatment significantly increased the levels of IL-6 and IL-8 to 4.60 pg/mL and 203.44 pg/mL, respectively. However, pretreatment with oat and oat bran polyphenols significantly inhibited the secretion of cytokines IL-6 and IL-8. The inhibitory effect of oat bran polyphenols was notably higher than that of oat polyphenols. Oat bound polyphenol and bran bound polyphenol reduced IL-6 levels by 73.07% and 84.04%, and IL-8 secretion levels by 50.56% and 55.53%, respectively, compared to DSS-induced model cells. While the inhibitory effect of bound polyphenol was higher than that of free phenols, the difference was not significant. This suggests that oat and bran polyphenols can protect the intestinal barrier from DSS and alleviate the inflammatory response induced by DSS.

### 3.6. Mitochondrial Membrane Potential

**Figure 5:**
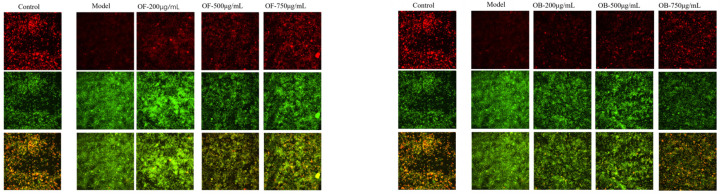

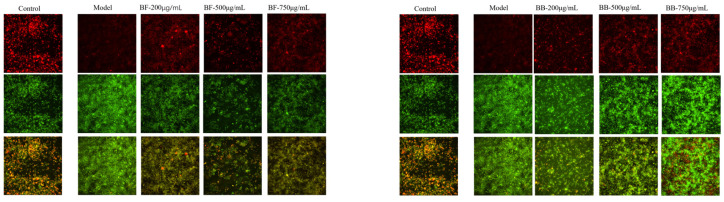
Effect of oat and bran polyphenols in LPS-induced mitochondrial membrane potential of Raw264.7 cells.

As shown in Figure 5, compared to the blank group, the fluorescence signals of JC-1 aggregates (red fluorescence) were significantly weakened, and the fluorescence signals of JC-1 monomers (green fluorescence) were significantly strengthened after 24 h of LPS stimulation. This indicates a significant decrease in cellular mitochondrial membrane potential. However, after treating the cells with oat and oat bran polyphenols, normal mitochondrial potential was restored.

### 3.7. Effect of Oat and Bran Polyphenols on the mRNA Expression of Inflammatory Factors

After LPS stimulation, the mRNA expression levels of all inflammatory mediators significantly increased in the cells, showing highly significant differences compared to the blank group. Specifically, TNF-α expression in the model group increased 2.24-fold compared to the blank group, IL-6 increased by 11.44-fold, iNOS increased 7.27-fold, COX-2 increased 4.24-fold, TLR4 increased 2.13-fold, and IL-10 increased 2.80-fold.

**Figure 6:**
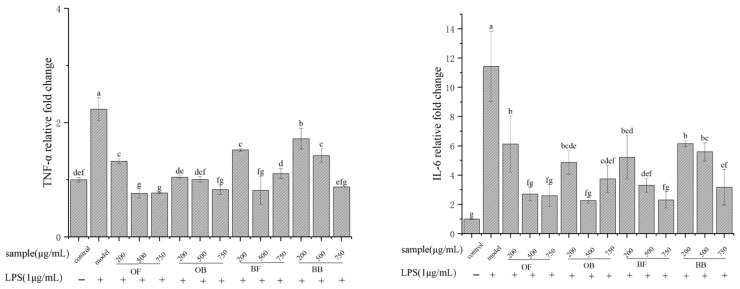

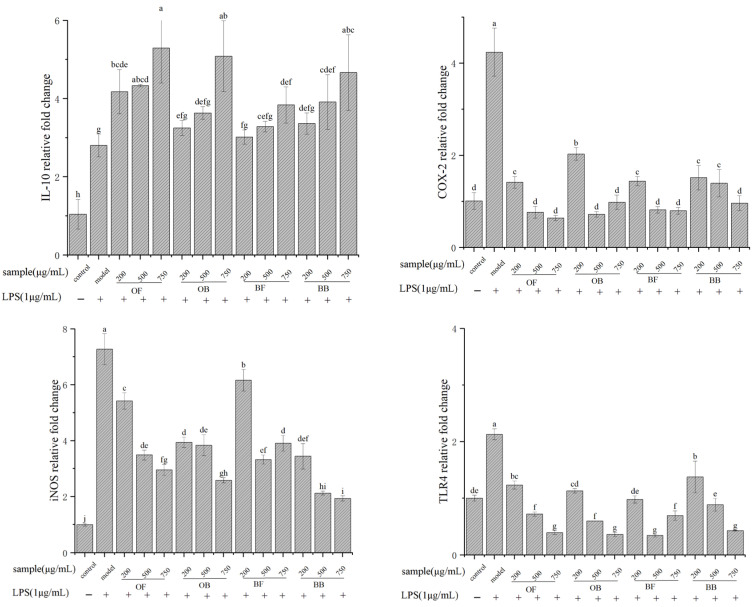
Effects of oat and bran polyphenol on the mRNA expression of TNF- α, IL-6, IL-10, COX-2, iNOS, and TLR4in Raw264.7 cells. Different lowercase represent significant differences among groups.

The effects of oat and oat bran polyphenols on the relative expression of IL-6, TNF-α, and IL-10, inflammation-related enzymes COX-2 and iNOS, as well as the cell surface receptor TLR4, were measured by RT-qPCR. In Figure 6, after LPS stimulation, the mRNA expression levels of all inflammatory mediators significantly increased in the cells, showing highly significant differences compared to the blank group. Specifically, TNF-α expression in the model group increased 2.24-fold compared to the blank group, IL-6 increased 11.44-fold, iNOS increased 7.27-fold, COX-2 increased 4.24-fold, TLR4 increased 2.13-fold, and IL-10 increased 2.80-fold. Compared to the model group, the relative gene expression of TNF-α and IL-6 was significantly decreased after cells were treated with oat and oat bran polyphenols. Oat and oat bran polyphenols significantly increased the relative expression of IL-10 genes after 24 h of LPS induction. After 24 h of LPS induction, high-dose oat and oat bran polyphenol (750 μg/mL) pretreatment had a significant inhibitory effect on the expression of COX-2, iNOS and TLR4 genes, but bran free phenol (500 μg/mL) pretreatment prompted a significant increase in the relative expression of iNOS and TLR4 genes compared with that of the high-dose (750 μg/mL) group.

### 3.8. Protection of the Intestinal Barrier by the Oat and Oat Bran Polyphenols

**Figure 7:**
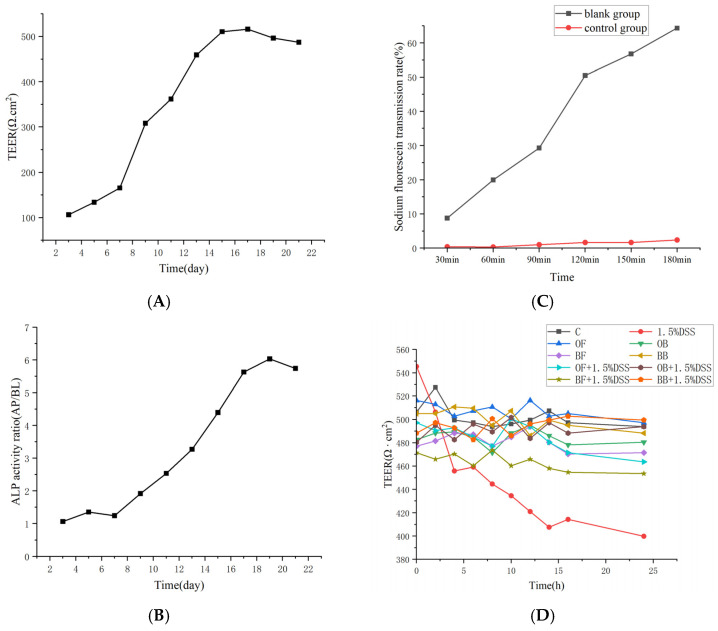
Change in TEER during the establishment of the Caco-2 monolayer (**A**), alkaline phosphatase activity (**B**), sodium fluorescein transmission rate (**C**), and TEER treated with 1.5% DSS and then with oat and bran polyphenols concentrations (1 mg/mL) to 24 h (**D**).

A Caco-2 cell monolayer model was used to mimic the intestinal epithelial cell barrier. To induce inflammation, differentiated Caco-2 cells were pretreated with oat and oat bran polyphenols for 24 h and then induced with 1.5% DSS for another 24 h to test their protective effect on the intestinal barrier. Figure 7A–C display the TEER values, ALP activity ratio (AP/BL), and sodium fluorescein transmission rate of the Caco-2 cells over 21 days during the establishment of the monolayer model. As the cells were incubated in the Transwell plate over time, the TEER value increased to 487.30 Ω · cm^2^, and the ALP activity ratio (AP/BL) gradually rose to 5.74 by the 21st day. The TEER value of the Caco-2 cells increased more rapidly during the initial 12 days of culture, slowed between days 12 to 16, and remained nearly constant from days 16 to 22. The sodium fluorescein transmittance rate was rapid in the blank group, while it was only 2.39% in the control group over 180 min. This indicated that after 21 days of culture, the Caco-2 cells had formed a dense monolayer, demonstrating good integrity of the cellular monolayer barrier. Thus, the in vitro model of the intestinal barrier was successfully established for subsequent experiments.

In Figure 7D, the resistance values of oat and bran polyphenols alone remained essentially unchanged after treating the cells for 24 h and did not affect the permeability of the cell membranes, and the addition of 1.5% DSS alone resulted in a drastic decrease in the TEER values from 0 to 12 h, and with the extension of the time, the resistance values eventually leveled off,, and the increase in intestinal permeability, indicating that the DSS disrupted the structure of the epithelial cellular barriers. Compared to the model group, administration of 1 mg/mL oat and bran polyphenols significantly attenuated the DSS-induced increase in cell barrier permeability, thereby protecting the cells from DSS-induced barrier damage. Bound polyphenol exhibited better protective effects in this regard.

### 3.9. Effect of Oat and Oat Bran Polyphenols on Intestinal Epithelial Barrier Genes

**Figure 8:**
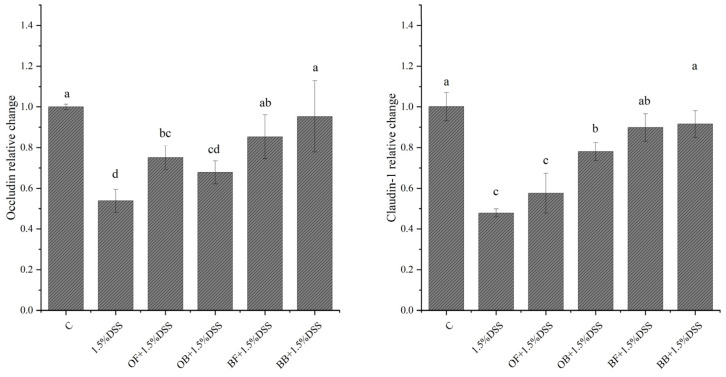
Effects of oat and bran polyphenol on the expression of Occludin and Claudin-1gene in DSS-induced Caco-2 cells. Different lowercase represent significant differences among groups.

To further investigate the molecular mechanisms by which oat and bran polyphenols protect epithelial barrier function, we examined the mRNA expression levels of tight junction proteins (Occludin and Claudin-1) in Caco-2 monolayer cells. In Figure 8, the gene expression levels of Occludin and Claudin-1 were significantly reduced after 1.5% DSS stimulation compared to the normal group, indicating that DSS disrupted the integrity of the intestinal epithelial barrier by decreasing Occludin and Claudin-1 gene expression. Compared to the model group, Claudin-1 expression was increased 1.63-fold by oat bound polyphenol, and 1.88-fold and 1.91-fold by bran free phenol and bound polyphenol, respectively. Additionally, the gene expression level in the bound-polyphenol group was higher than in the free-polyphenol group. In contrast, compared to the model group, oat bound phenol, oat bran free polyphenol, and oat bran bound polyphenol treatment groups showed significantly higher Occludin gene expression levels. Oat free phenol and bound polyphenol increased Occludin gene expression 1.39-fold and 1.26-fold, respectively, while bran free phenol and bound polyphenol increased Occludin gene expression 1.59-fold and 1.77-fold, respectively. This suggests that oat and bran polyphenols can promote Occludin and Claudin-1 gene expression in cells, thereby protecting the intestinal epithelial barrier function.

## 4. Discussion

Phenolic acids of most cereals are concentrated in bran and germ, which are divided into free and bound states, and mainly in the bound state. The bound phenolic acids are connected with the main components of cell wall, such as protein and cellulose, in the form of ester bonds. Gong et al. (2019) [32] showed that the content of free phenols and free flavonoids in cereals is lower than that of bound polyphenol and bound flavonoids, which is consistent with the experimental results. Ferulic acid is mainly distributed in conjugated phenols. Gong et al. [32] reported that ferulic acid is also the main phenolic substance in oats, and this consistent with our study. Adom et al. (2002) [11] similarly found that 98% of ferulic acid in cereals was present in the bound polyphenol. Of course, because oat alkaloids are easy to degrade under strong alkaline conditions, we only measured free polyphenols. Hu et al. (2020) [33] showed that the total alkaloid content of six commercial sprouted oats was from 7.85 to 133.3 μg/g. Through comparative analysis, the content of alkaloid C in oats grown in European and American countries is generally higher than that in oats tested in this experiment (1.33–8.82 μg/g DW), which may be related to the growth environment, geographical location and extraction method of oats. Consistent with our experimental results, Hitayezu et al. (2015) [34] showed that the content of alkaloids in oats gradually decreased from outer bran to aleurone layer and then to endosperm, the content of avenanthramides C in bran was significantly higher than oats.

Macrophages are the first line of defense of the body’s immune response, with the functions of recognizing, phagocytosing, and removing bacteria and foreign objects. However, when macrophages are abnormally activated, they can secrete large amounts of pro-inflammatory cytokines, playing an important role in immune system homeostasis, and regulating excessive inflammation is essential for recovery of body functions [35,36]. The tight junction of intestinal epithelial cells is closely related to the development of various intestinal-related diseases such as impaired intestinal mucosal barrier function [7]. Therefore, the establishment of in vitro models of inflammation in macrophages and intestinal epithelial cells is necessary for studying the anti-inflammatory effects of drugs.

In our study, we assessed the potential of oat and oat bran polyphenols to alleviate cellular damage induced by LPS-stimulated RAW264.7 macrophages and DSS-stimulated Caco-2 cells. Macrophage phagocytosis plays a crucial role in the body’s defense against pathogen infections. In addition, the effects of oat and bran polyphenols on phagocytic activity of RAW264.7 cells were determined by neutral red assay. The results revealed that LPS stimulation significantly enhanced the phagocytosis of RAW264.7 cells. However, pretreatment with oat and bran polyphenols inhibited LPS-induced phagocytosis, indicating that oat and bran polyphenols enhance the cells’ anti-inflammatory capacity by modulating their phagocytic function and thereby protect the organism against antigenic infections.

It has been shown that macrophages stimulated by LPS release large amounts of inflammatory mediators such as NO and ROS. NO is a cytokine that maintains immunity and participates in various physiological processes within the organism. Excessive NO secretion during inflammation can lead to cell death [37,38,39]. ROS, as the primary mediator of cellular oxidative stress responses, can damage mitochondria and exacerbate cellular damage and inflammatory responses by reducing mitochondrial membrane potential [40]. Studies have demonstrated that the bioavailability of active compounds in physiological systems is often significantly lower than under in vitro conditions, primarily due to limited aqueous solubility, poor in vivo absorption, and inadequate metabolic stability. This discrepancy poses challenges in translating concentrations effective in cellular or animal models to clinically relevant dosages. Moreover, conventional in vitro experimental paradigms tend to emphasize acute cellular interactions, often overlooking the effects of prolonged exposure and the role of post-metabolic mechanisms of action [41]. The concentration of 100 μg/mL did not significantly inhibit NO production, which may be due to insufficient concentration, resulting in an intracellular concentration below the threshold needed for effective action. Moreover, the anti-inflammatory effects of polyphenolic substances showed high variability depending on the source and extraction methods. Therefore, in this experiment, oat and bran polyphenols at concentrations ranging from 200 to 750 μg/mL significantly inhibited NO and ROS production in a concentration-dependent manner. The inhibitory effect on NO and ROS production was particularly pronounced at higher doses. Furthermore, the present study demonstrated that pretreatment with oat and oat bran polyphenols restored mitochondrial membrane potential.

In addition, activated inflammatory cells secrete inflammatory factors, such as TNF-α and IL-6, which further exacerbate the systemic inflammatory response. TNF-α plays a crucial role in the onset and progression of inflammation, promoting the synthesis and release of other inflammatory cytokines [42]. IL-6 is produced by a wide range of immune cells and is involved in regulating the majority of inflammation-induced acute phase proteins [43]. The results of this study showed that pretreatment with polyphenols from oats and oat bran significantly reduced the production of TNF-α and IL-6, while markedly increasing the level of IL-10, thereby further alleviating the inflammatory response exhibited by macrophages.

Existing studies have shown that the mechanism of LPS-induced inflammatory response in RAW 264.7 cells is mainly attributed to LPS binding to the cell surface receptor TLR4 and activating intracellular pathways related to inflammation, thereby promoting the secretion of large amounts of inflammatory factors by cells. Cell injury further activates key enzymes such as iNOS and COX-2, triggering an inflammatory response [44]. iNOS and COX-2 are key enzymes upstream of NO and PGE2 synthesis. Previous findings have shown that RAW264.7 cells stimulated by LPS express significant quantities of COX-2 and iNOS mRNA, leading to the production of substantial amounts of NO and PGE2 [45]. In this study, gene expression was measured, revealing that the expression level of the TLR4 in the model group was significantly higher than that in the blank group, suggesting that TLR4 activation promotes downstream inflammatory factor expression. The subsequent increase in IL-6 and TNF further activates the inflammatory pathway, exacerbating the inflammatory response. After pretreatment with oat and bran polyphenols, the expression of COX-2 and iNOS mRNA was significantly inhibited, reducing the synthesis and secretion of pro-inflammatory cytokines, and decreasing macrophage phagocytosis. This alleviated and inhibited the onset and progression of inflammation, indicating that oat and bran polyphenols exhibit pronounced anti-inflammatory effects [44].

The degree of change in cell membrane permeability can reflect the function of intercellular tight junctions and cell monolayer barrier. The intestinal epithelium is an important barrier that protects the body from external harmful substances [46]. TEER values accurately indicate the integrity and permeability of the epithelial barrier. Higher TEER values indicate better intestinal barrier integrity and lower permeability. In this study, to assess intestinal epithelial barrier function, we measured cell permeability by evaluating TEER values using sodium fluorescein and alkaline phosphatase activity. Treatment with DSS has been shown to damage intestinal cells and increase intestinal permeability, thereby impairing intestinal barrier function [47]. Abnormal expression of inflammatory cytokines such as IL-6 and IL-8 is a major cause of disrupted intercellular tight junctions in intestinal tissues and damage to the epithelial barrier. In this study, we observed that DSS-stimulated Caco-2 cells produced inflammatory factors IL-6 and IL-8, which led to decreased TEER values. Treatment with oat and bran polyphenols stabilized TEER values, mitigated hyperpermeability in the model group, and preserved the integrity of the cellular monolayer, thereby reducing inflammation in intestinal cells. Notably, the present study found that oat bran polyphenols exhibited toxicity at a concentration of 5 mg/mL in the Caco-2 cell model, while showing cytoprotective effects at lower concentrations. This finding is consistent with previous studies, which indicate that dietary polyphenols have cytoprotective effects at low doses. However, at high doses, they may cause toxic side effects, leading to negative outcomes [48].

Occludin and Claudin-1 are structurally important proteins that constitute tight junctions (TJs) and are major protein molecules that maintain intestinal epithelial barrier function and determine intestinal permeability [49]. Our results indicate that DSS-induced damage to the epithelial barrier was reversed by both oat and oat bran polyphenols treatments, and the anti-inflammatory effects of oat and bran polyphenols in intestinal cells Caco-2 are mainly reflected in the alleviation of DSS-induced intestinal barrier disruption by decreasing pro-inflammatory cytokine release and increasing the expression of tight junctions proteins, thus enhancing the intestinal mucosal barrier function.

## 5. Limitations

It should be noted that this study did not model the potential effects of the digestion process on the structure and anti-inflammatory activity of polyphenols. Future research will incorporate in vitro digestion models, such as the INFOGEST protocol, to more accurately evaluate the bioavailability and biological activity of oat-derived polyphenols in vivo.

## 6. Conclusions

Based on an in vitro cellular inflammation model, we explored the anti-inflammatory activities of oat and bran polyphenols. The results demonstrated that the extract exhibited anti-inflammatory activity within the effective concentration range of 200–750 μg/mL. Oat and oat bran polyphenols effectively inhibited NO and ROS production, suppressed the secretion of IL-6 and TNF-α inflammatory factors, increased mitochondrial membrane potential, and down-regulated the expression of inflammation-related genes TNF-α and IL-6. These actions could reduce organismal damage and exert anti-inflammatory effects. In the Caco-2 intestinal cell barrier model stimulated with DSS, oat and bran polyphenol treatments increased the mRNA expression levels of Claudin-1 and Occludin proteins in Caco-2 cells, thereby exerting a protective effect on intestinal barrier function. The study initially explored the in vitro anti-inflammatory activity of oat and bran polyphenols. Although its efficacy in vivo remains to be further validated, this is particularly important given that many polyphenols may exhibit low absorption and bioavailability. Therefore, follow-up studies are needed to further validate these findings using Caco-2 cell transporter assays or dynamic digestion models. Based on the current experimental results, future research will include the evaluation of the pharmacokinetic profile in animal models and the validation of in vivo anti-inflammatory effects at doses approximating dietary intake, and relevant data will be published in subsequent work. Additionally, we plan to establish a Caco-2/RAW 264.7 co-culture system in a follow-up study to dynamically analyze the trans-barrier transport of oat polyphenols and their modulation of macrophage inflammation. It is also important to determine whether a single component or a mixture of components is responsible for their observed effects. This study confirms that oat and bran polyphenol extracts exhibit anti-inflammatory effects; however, the potential influence of other phytochemicals on their bioavailability requires further in-depth investigation.

## Data Availability

The raw data supporting the conclusions of this article will be made available by the authors on request.

## Appendix A

**Table A1:** Contents of common phenolic components of oat and oat bran.

Samples	Part	Phytochemicals (μg/g DW)							
		Gallic Acid	Protocatechuic Acid	p-Hydroxybenzoic Acid	Vanillic Acid	Caffeic Acid	p-Coumaric Acid	Ferulic Acid	Avenanthramides C
Oat	Free	2.72 ± 0.04	0.52 ± 0.01	0.92 ± 0.01	1.2 ± 0.12	6.86 ± 0.17	1.15 ± 0.14	5.26 ± 0.02	1.33 ± 0.19
	Bound	ND	5.73 ± 0.03	8.32 ± 0.03	1.47 ± 0.09	0.86 ± 0.05	0.75 ± 0.74	198.09 ± 0.76	ND
	Total	2.72 ± 0.04	6.24 ± 0.03	9.24 ± 0.03	2.67 ± 0.08	7.71 ± 0.14	1.91 ± 0.88	203.36 ± 0.74	1.33 ± 0.19
Oat Bran	Free	6.48 ± 0.02	1.13 ± 0.01	7.82 ± 0.03	0.93 ± 0.01	1.22 ± 0	2.91 ± 0.14	11.28 ± 0.02	8.82 ± 4.04
	Bound	ND	10.49 ± 0.05	8.03 ± 0.07	0.29 ± 0.04	1.76 ± 0.06	2.92 ± 0.11	335.15 ± 1.64	ND
	Total	6.48 ± 0.02	11.62 ± 0.04	15.85 ± 0.09	1.22 ± 0.04	2.98 ± 0.06	5.83 ± 0.19	346.43 ± 1.62	8.82 ± 4.04

Notes: ND: not detected.

## References

[1] Kellow N.J., Coughlan M.T. (2015). Effect of diet-derived advanced glycation end products on inflammation. Nutr. Rev..

[2] Fredman G. (2019). DELineating resolution of inflammation. Nat. Immunol..

[3] Grant W.B. (2023). Diet, Inflammation, and infectious diseases. Nutrients.

[4] Zhang Z., Tanaka I., Pan Z., Ernst P.B., Kiyono H., Kurashima Y. (2022). Intestinal homeostasis and inflammation: Gut microbiota at the crossroads of pancreas–intestinal barrier axis. Eur. J. Immunol..

[5] Martins-Gomes C., Nunes F.M., Silva A.M. (2024). Natural products as dietary agents for the prevention and mitigation of oxidative damage and inflammation in the intestinal barrier. Antioxidants.

[6] Kaplan G.G., Ng S.C. (2017). Understanding and preventing the global increase of inflammatory bowel disease. Gastroenterology.

[7] Davis E.M., Kaufmann Y., Goyne H., Wang Y.X., Chen T., Theus S., Liu J.J. (2017). Pyroptosis of intestinal epithelial cells is crucial to the development of mucosal barrier dysfunction and intestinal inflammation. Gastroenterology.

[8] Song X., Zhang X., Ma C., Hu X., Chen F. (2022). Rediscovering the nutrition of whole foods: The emerging role of gut microbiota. Curr. Opin. Food Sci..

[9] Fang W., Peng W., Qi W., Zhang J., Song G., Pang S., Wang Y. (2024). Ferulic acid combined with different dietary fibers improve glucose metabolism and intestinal barrier function by regulating gut microbiota in high-fat diet-fed mice. J. Funct. Foods.

[10] Roager H.M., Vogt J.K., Kristensen M., Hansen L.B.S., Ibrügger S., Mærkedahl R.B., Licht T.R. (2019). Whole grain-rich diet reduces body weight and systemic low-grade inflammation without inducing major changes of the gut microbiome: A randomised cross-over trial. Gut.

[11] Adom K.K., Liu R.H. (2002). Antioxidant activity of grains. J. Agric. Food Chem..

[12] Awika J.M., Rose D.J., Simsek S. (2018). Complementary effects of cereal and pulse polyphenols and dietary fiber on chronic inflammation and gut health. Food Funct..

[13] Gao H., Song R.J., Jiang H., Zhang W., Han S.F. (2022). Oat fiber supplementation alleviates intestinal inflammation and ameliorates intestinal mucosal barrier via acting on gut microbiota-derived metabolites in LDLR^–/–^ mice. Nutrition.

[14] Dong L., Qin C., Li Y., Wu Z., Liu L. (2022). Oat phenolic compounds regulate metabolic syndrome in high fat diet-fed mice via gut microbiota. Food Biosci..

[15] Ma L., Luo Z., Huang Y., Li Y., Guan J., Zhou T., Cao S. (2022). Modulating gut microbiota and metabolites with dietary fiber oat β-glucan interventions to improve growth performance and intestinal function in weaned rabbits. Front. Microbiol..

[16] Soycan G., Schär M.Y., Kristek A., Boberska J., Alsharif S.N.S., Corona G., Spencer J.P.E. (2019). Composition and content of phenolic acids and avenanthramides in commercial oat products: Are oats an important polyphenol source for consumers?. Food Chem. X.

[17] Zhang L., Wu T., Zhang Y., Chen Y., Ge X., Sui W., Zhang M. (2023). Release of bound polyphenols from wheat bran soluble dietary fiber during simulated gastrointestinal digestion and colonic fermentation in vitro. Food Chem..

[18] Li J., Zhang H., Yang X., Zhu L., Wu G., Qi X., Zhang H. (2022). Trapping of reactive carbonyl species by fiber-bound polyphenols from whole grains under simulated physiological conditions. Food Res. Int..

[19] Chen C., Wang L., Chen Z.X., Luo X.H., Li Y.F., Wang R., Li J., Li Y.A., Wang T., Wu J. (2019). Effects of milk proteins on the bioaccessibility and antioxidant activity of oat phenolics during in vitro digestion. J. Food Sci..

[20] Li X., Du Y., Zhang C., Tu Z., Wang L. (2022). Modified highland barley regulates lipid metabolism, liver inflammation and gut microbiota in high-fat/cholesterol diet mice as revealed by LC-MS based metabonomics. Food Funct..

[21] Hong S., Pangloli P., Perumal R., Cox S., Noronha L.E., Dia V.P., Smolensky D. (2020). A comparative study on phenolic content, antioxidant activity and anti-inflammatory capacity of aqueous and ethanolic extracts of sorghum in lipopolysaccharide-induced RAW 264.7 macrophages. Antioxidants.

[22] Yang R., Shan S., Zhang C., Shi J., Li H., Li Z. (2020). Inhibitory effects of bound polyphenol from foxtail millet bran on colitis-associated carcinogenesis by the restoration of gut microbiota in a mice model. J. Agric. Food Chem..

[23] Chen S.W., Zhu J., Zuo S., Zhang J., Chen Z.Y., Chen G.W., Wang P. (2015). Protective effect of hydrogen sulfide on TNF-α and IFN-γ-induced injury of intestinal epithelial barrier function in Caco-2 monolayers. Inflamm. Res..

[24] Kim K.J., Kim Y., Jin S.G., Kim J.Y. (2021). Acai berry extract as a regulator of intestinal inflammation pathways in a Caco-2 and RAW 264.7 co-culture model. J. Food Biochem..

[25] He Z.Q., Deng N., Zheng B.S., Li T., Liu R.H., Yuan L., Li W.Z. (2022). Changes in polyphenol fractions and bacterial composition after in vitrofermentation of apple peel polyphenol by gut microbiota. Int. J. Food Sci. Technol..

[26] Ji K.-Y., Kim K.M., Kim Y.H., Im A.R., Lee J.Y., Park B., Chae S. (2019). The enhancing immune response and anti-inflammatory effects of Anemarrhena asphodeloides extract in RAW 264.7 cells. Phytomedicine.

[27] Xiong L., Ouyang K.H., Jiang Y., Yang Z.W., Hu W.-B., Chen H., Wang W.J. (2018). Chemical composition of Cyclocarya paliurus polysaccharide and inflammatory effects in lipopolysaccharide-stimulated RAW264.7 macrophage. Int. J. Biol. Macromol..

[28] Wang H., Guo X., Liu J., Li T., Fu X., Liu R.H. (2017). Comparative suppression of NLRP3 inflammasome activation with LPS-induced inflammation by blueberry extracts (*Vaccinium* spp.). RSC Adv..

[29] Kapoor S., Padwad Y.S. (2023). Phloretin suppresses intestinal inflammation and maintained epithelial tight junction integrity by modulating cytokines secretion in model of gut inflammation. Cell. Immunol..

[30] Yang Y., Ren R.R., Chen Q.Q., Zhang Q.Q., Wu J.J., Yin D.K. (2022). Coptis chinensis polysaccharides dynamically influence the paracellular absorption pathway in the small intestine by modulating the intestinal mucosal immunity microenvironment. Phytomedicine.

[31] Guo T.Y., Lin Q.L., Li X.H., Nie Y., Wang L., Shi L.M., Luo F.J. (2017). Octacosanol attenuates inflammation in both RAW264.7 macrophages and a mouse model of colitis. J. Agric. Food Chem..

[32] Gong E.S., Gao N.X., Li T., Chen H.Y., Wan Y.H., Si X., Liu R.H. (2019). Effect of in vitro digestion on phytochemical profiles and cellular antioxidant activity of whole grains. J. Agric. Food Chem..

[33] Hu C.L., Tang Y., Zhao Y.T., Sang S.M. (2020). Quantitative analysis and anti-inflammatory activity evaluation of the a-type avenanthramides in commercial sprouted oat products. J. Agric. Food Chem..

[34] Hitayezu R., Baakdah M.M., Kinnin J., Henderson K., Tsopmo A. (2015). Antioxidant activity, avenanthramide and phenolic acid contents of oat milling fractions. J. Cereal Sci..

[35] Schultze J.L., Rosenstiel P., Consortium S. (2018). Systems medicine in chronic inflammatory diseases. Immunity.

[36] Van den Bossche J., Baardman J., Otto N.A., van der Velden S., Neele A.E., van den Berg S.M., de Winther M.P.J. (2016). Mitochondrial dysfunction prevents repolarization of inflammatory macrophages. Cell Rep..

[37] Guo C., Yang L., Luo J., Zhang C., Xia Y.Z., Ma T., Kong L.Y. (2016). Sophoraflavanone G from inhibits lipopolysaccharide-induced inflammation in RAW264.7 cells by targeting PI3K/Akt, JAK/STAT and Nrf2/HO-1 pathways. Int. Immunopharmacol..

[38] Hsieh S.C., Hsieh W.J., Chiang A.N., Su N.W., Yeh Y.T., Liao Y.C. (2016). The methanol-ethyl acetate partitioned fraction from Chinese olive fruits inhibits cancer cell proliferation and tumor growth by promoting apoptosis through the suppression of the NF-κB signaling pathway. Food Funct..

[39] Nathan C., Xie Q.W. (1994). Nitric oxide synthases: Roles, tolls, and controls. Cell.

[40] Ishii M., Nakahara T., Araho D., Murakami J., Nishimura M. (2017). Glycolipids from spinach suppress LPS-induced vascular inflammation through eNOS and NK-κB signaling. Biomed. Pharmacother..

[41] Yang B.Y., Dong Y.X., Wang F., Zhang Y. (2020). Nanoformulations to enhance the bioavailability and physiological functions of polyphenols. Molecules.

[42] Wang S.Y., Shi X.J., Li J., Huang Q.P., Ji Q., Yao Y., Yang G. (2022). A small molecule selected from a DNA-Encoded library of natural products that binds to TNF-α and attenuates inflammation in vivo. Adv. Sci..

[43] Ye M., Joosse M.E., Liu L., Sun Y., Dong Y., Cai C.C., Li X.H. (2020). Deletion of IL-6 exacerbates colitis and induces systemic inflammation in IL-10-deficient mice. J. Crohn’s Colitis.

[44] He J., Han S., Li X.X., Wang Q.Q., Cui Y., Chen Y., Yang S. (2019). Diethyl blechnic exhibits anti-Inflammatory and antioxidative activity via the TLR4/MyD88 signaling pathway in LPS-stimulated RAW264.7 cells. Molecules.

[45] George G., Shyni G.L., Abraham B., Nisha P., Raghu K.G. (2021). Downregulation of TLR4/MyD88/p38MAPK and JAK/STAT pathway in RAW 264.7 cells by Alpinia galanga reveals its beneficial effects in inflammation. J. Ethnopharmacol..

[46] Jutanom M., Kato S., Yamashita S., Toda M., Kinoshita M., Nakagawa K. (2023). Analysis of oxidized glucosylceramide and its effects on altering gene expressions of inflammation induced by LPS in intestinal tract cell models. Sci. Rep..

[47] Chen Y.H., Shin J.Y., Wei H.M., Lin C.C., Yu L.C.H., Liao W.T., Chu C.L. (2021). Prevention of dextran sulfate sodium-induced mouse colitis by the fungal protein Ling Zhi-8 promoting the barrier function of intestinal epithelial cells. Food Funct..

[48] Calabrese V., Cornelius C., Trovato-Salinaro A., Cambria M.T., Locascio M.S., Di Rienzo L., Condorelli D.F., Mancuso C., De Lorenzo A., Calabrese E.J. (2010). The hormetic role of dietary antioxidants in free radical-related diseases. Curr. Pharm. Des..

[49] Bein A., Zilbershtein A., Golosovsky M., Davidov D., Schwartz B. (2017). LPS induces hyper-permeability of intestinal epithelial cells. J. Cell. Physiol..

